# Biofluid quantification of TWEAK/Fn14 axis in combination with a selected biomarker panel improves assessment of prostate cancer aggressiveness

**DOI:** 10.1186/s12967-019-2053-6

**Published:** 2019-09-09

**Authors:** Xavier Ruiz-Plazas, Esther Rodríguez-Gallego, Marta Alves, Antonio Altuna-Coy, Javier Lozano-Bartolomé, Manel Portero-Otin, Joan Francesc García-Fontgivell, Salomé Martínez-González, José Segarra, Matilde R. Chacón

**Affiliations:** 1 Disease Biomarkers and Molecular Mechanisms Group, IISPV, Joan XXIII University Hospital, Universitat Rovira i Virgili, University Hospital of Tarragona Joan XXIII, C/Dr. Mallafré Guasch, 4, 43007 Tarragona, Spain; 2 Urology Unit, Joan XXIII University Hospital, Tarragona, Spain; 3 Infectious Diseases and HIV/AIDS Unit, Department of Internal Medicine, Joan XXIII University Hospital, Universitat Rovira i Virgili, Tarragona, Spain; 4 Department of Experimental Medicine, Universitat de Lleida-IRBLleida, Lleida, Spain; 5 Pathology Unit, Joan XXIII University Hospital, Tarragona, Spain

**Keywords:** TWEAK/Fn14 axis, Prostate cancer, Biomarkers, Diagnosis, Biofluids

## Abstract

**Background:**

Conventional clinical biomarkers cannot accurately differentiate indolent from aggressive prostate cancer (PCa). We investigated the usefulness of a biomarker panel measured exclusively in biofluids for assessment of PCa aggressiveness.

**Methods:**

We collected biofluid samples (plasma/serum/semen/post-prostatic massage urine) from 98 patients that had undergone radical prostatectomy. Clinical biochemistry was performed and several cytokines/chemokines including soluble(s) TWEAK, sFn14, sCD163, sCXCL5 and sCCL7 were quantified by ELISA in selected biofluids. Also, the expression of *KLK2*, *KLK3*, *Fn14*, *CD163*, *CXCR2* and *CCR3* was quantified by real-time PCR in semen cell sediment. Univariate, logistic regression, and receiver operating characteristic (ROC) analyses were used to assess the predictive ability of the selected biomarker panel in conjunction with clinical and metabolic variables for the evaluation of PCa aggressiveness.

**Results:**

Total serum levels of prostate-specific antigen (PSA), semen levels of sTWEAK, fasting glycemia and mRNA levels of *Fn14*, *KLK2*, *CXCR2* and *CCR3* in semen cell sediment constituted a panel of markers that was significantly different between patients with less aggressive tumors [International Society of Urological Pathology (ISUP) grade I and II] and those with more aggressive tumors (ISUP grade III, IV and V). ROC curve analysis showed that this panel could be used to correctly classify tumor aggressiveness in 90.9% of patients. Area under the curve (AUC) analysis revealed that this combination was more accurate [AUC = 0.913 95% confidence interval (CI) 0.782–1] than a classical non-invasive selected clinical panel comprising age, tumor clinical stage (T-classification) and total serum PSA (AUC = 0.721 95% CI 0.613–0.830).

**Conclusions:**

TWEAK/Fn14 axis in combination with a selected non-invasive biomarker panel, including conventional clinical biochemistry, can improve the predictive power of serum PSA levels and could be used to classify PCa aggressiveness.

## Background

Prostate cancer (PCa) is the fourth most common cancer in both sexes collectively and the second most common cancer in men [1]. PCa is a curable disease if detected at an early stage and there are several viable therapeutic options available; however, many patients fail to respond to first-line treatments. Conventional clinical prognosticators of PCa severity and progression include digital rectal examination, serum prostate-specific antigen (PSA) measurement, and transrectal ultrasound-guided histopathological staging [2]. PSA is encoded in humans by the kallikrein-3 (*KLK3*) gene [3] and its secreted protein is a pre-propeptide that can be activated by human glandular kallikrein-2 protease, encoded by *KLK2* [4]. While the application of PSA serum testing as a screening tool for PCa has reduced mortality rates and the prevalence of advanced-stage disease at diagnosis, there is continuing debate over its efficacy as a specific marker for diagnosis and prognosis [5]. Accordingly, robust biomarkers are needed for the early screening of patients at risk of PCa.

Liquid biopsy is a promising non-invasive modality for molecular profiling in cancer, enabling the assessment of circulating molecules in various biological fluids for biomarker discovery [6]. Moreover, when combined with multi-marker analysis, liquid biopsy is a potentially powerful platform for prognosis and early assessment of treatment failure.

TNF-like weak inducer of apoptosis (TWEAK) and fibroblast growth factor-inducible molecule 14 (Fn14) are a ligand/receptor pair belonging to the TNF receptor superfamily. TWEAK is synthesized as a membrane-anchored protein (mTWEAK) that is released into circulation in a soluble form (sTWEAK) after processing by a furin protease. Both sTWEAK and mTWEAK function through engagement with Fn14 [7]. TWEAK has pleiotropic effects on different cell functions, mediating pro-inflammatory and pro-angiogenic activity, and stimulating invasion, migration and survival [8, 9]. Fn14 is expressed at relatively low levels in normal tissues, but is significantly upregulated locally following tissue injury or disease, where it mediates tissue remodeling [7]. Interestingly, Fn14 overexpression promotes androgen-independent PCa progression and correlates with poor treatment outcome [10]. Many TNF receptors undergo proteolytic cleavage to generate soluble forms that are shed into biofluids, and a recent study reported the presence of high levels of soluble Fn14 (sFn14) in urine samples of patients with kidney injury [11], pointing to sFn14 as a potential biomarker for progression of kidney disease.

In addition to binding Fn14, sTWEAK can also bind to the CD163 (cluster of differentiation 163) receptor, which acts as a scavenger receptor since its binding does not transduce intracellular signals but instead mediates the elimination of sTWEAK, contributing to its degradation [12]. CD163 is expressed exclusively on monocytes/macrophages and is tightly regulated by inflammatory responses and anti-inflammatory signals [13]. Similar to Fn14, CD163 not only exists as a membrane-bound form, but is also released into circulation as a soluble form (sCD163), and is a sensitive indicator of macrophage activation [14]. Interestingly, the presence of CD163^+^ macrophages is strongly associated with less favorable clinical and pathological features in breast cancer [15].

Several chemokines are known to play key roles in the development and progression of PCa [16]. For example, the apoptosis-induced chemokine CXCL5 and its receptor CXCR2 have been reported to accelerate inflammation and growth of prostate tumor metastases in bone [17]. Moreover, CXCL5/CXCR2 expression was found to correlate with Gleason grading and clinical pathologic stages [18]. Another study examined the CCL7/CCR3 axis in a mouse model of PCa and obesity, specifically in the periprostatic adipose tissue surrounding the tumor, finding that this tissue secretes CCL7, which in turn stimulates the migration of CCR3-expressing tumor cells [19]. Notably, depletion of tumor CCR3 completely abolished the ability of obesity to promote tumor metastasis [15]. Crosstalk between CCL7 and CCR3 has also been found to promote colon cancer metastasis [20].

Because the aforementioned biomolecules, TWEAK, CXCL5 and CCL7 and their respective receptors (Fn14, CXCR2 and CCR3) as well as PSA-related genes (*KLK3* and *KLK2*) are known to be linked to tumorigenesis and could therefore provide a growth advantage to tumors, we designed a study to quantify and examine these selected biomarkers in several biofluids from patients with PCa, to test whether they could predict tumor aggressiveness.

## Materials and methods

### Patients and study design

This retrospective study included 98 consecutive patients with PCa who had undergone radical prostatectomy by open surgery—laparoscopic or robotic surgery (intraperitoneal or extraperitoneal)—with or without bilateral ilio-obturator lymphadenectomy, according to estimated risk of lymphadenopathy based on the Briganti nomogram [21]. Surgeries were performed at the University Hospital Joan XXIII, Tarragona, between 2015 and 2018. Grades, groups and stage of the tumors were determined according to the 2014 International Society of Urological Pathology (ISUP) Gleason Grading (GG) and Tumor Node Metastases (TNM) classification, respectively [22, 23]. We stratified patients according to ISUP GG into two categories: low-risk (ISUP group I and II) and high-risk (ISUP groups III, IV and V).

The study was performed according to the provisions of the Declaration of Helsinki, was approved by our local ethics committee, and adhered to current legal regulations (Biomedical Research Law 14/2007, Royal Decree of Biobanks 1716/2011, Organic Law 15/1999 of September 13 Protection of Personal Data). All participants provided written informed consent prior to their inclusion. Clinical parameters regarding tumor aggressiveness and metabolic status of all patients were recorded. All methods were performed in accordance with the relevant guidelines and regulations. Inclusion criteria were as follows: patients older than 18 years diagnosed with PCa by prostate biopsy in our center or any other, treated by radical prostatectomy in our center, and with signed informed consent for the study. Exclusion criteria were patients with previous history of cancer, patients older than 75 years, and those who had received any prior treatment before radical prostatectomy for PCa. Body mass index (BMI) was measured using standard procedures and World Health Organization criteria.

### Biofluid processing

#### Post digital rectal examination urine

First-catch voided urine (30 mL) was collected after attentive digital rectal examination and prior to prostate biopsy or surgical intervention. Urine samples were immediately centrifuged (2000×*g*, 10 min, 4 °C), and stored at − 80 °C for further examination.

#### Serum

After a fast of at least 12 h, blood was obtained from the antecubital vein. Samples were centrifuged for 15 min at 5000×*g* and stored at − 80 °C for further determinations. All clinical biochemistry variables were calculated according to standardized methods. The homeostatic model assessment-insulin resistance index (HOMA-IR) was calculated using the formula: (glucose [mmol/L] × insulin [mU L^−1^])/22.5).

#### Semen

Ejaculate specimens from 52 patients were centrifuged at 2000×*g* for 15 min at 22 °C to separate spermatozoa from semen plasma. The supernatant (semen plasma) was then aliquoted into 100 µL aliquots and frozen at − 80 °C until further processing. The cell sediment was washed once with one volume of phosphate buffered saline and stored frozen at − 80 °C until further RNA extraction.

### Enzyme-linked immunosorbent assays

Levels of sTWEAK, sCD163, sCXCL5, sCCL7 in biofluids were determined in duplicate using commercially available human enzyme-linked immunosorbent assay (ELISA) DuoSet Kits (R&D Systems Europe, Abingdon, UK). For sFn14 quantification, we used commercial ELISA kits from Aviva Systems Biology (Bionova Cientifica, Barcelona, Spain). Urine biomarker levels were normalized to creatinine urine levels, whereas semen biomarker levels were normalized to total protein measured by the bicinchoninic acid assay method (Pierce™ BCA Protein Assay Kit, Thermo Fisher Scientific, Barcelona, Spain).

### Quantitative reverse transcription-polymerase chain reaction

RNA from semen cell sediments was extracted using TRIzol reagent (Invitrogen, Carlsbad, CA, USA) and quantified with a NanoDrop device (NanoDrop Technologies, Wilmington, DE, USA). cDNA was synthesized from 500 ng of total RNA using the High Capacity cDNA reverse transcription kit (Applied Biosystems, Foster City, CA, USA). Real-time quantitative PCR was performed on a 7900HT fast real-time PCR system using commercial Taqman assays (Applied Biosystems) for the following genes: *Fn14*, *CD163*, *KLK2*, *KLK3*, *CXCR2* and *CCR3*. The cycle threshold (C_t_) value for each sample was normalized to the expression of *PPIA* (cyclophilin A), which showed no significant gene expression differences between the compared groups of patients. SDS software 2.3 and RQ Manager 1.2 (Applied Biosystems) were used to analyze the results with the comparative C_t_ method (2^−∆∆Ct^). All data were expressed as an n-fold difference relative to the calibrator (a mixture of the RNA from 4 different patients was used as a calibrator sample).

### Statistical analysis

For clinical and anthropometrical variables, data are expressed as mean ± standard deviation (SD). Before statistical analysis, normal distribution and homogeneity of the variances was evaluated using Levene’s test, and then variables were log-transformed when necessary. Differences between patients according to ISUP GG—low-risk (Group I and II) and high-risk ISUP GG (Group III, IV and V)—were tested with the Mann–Whitney U test for non-normally distributed data. A *p*-value less than 0.05 was considered statistically significant. Logistic regression analysis and receiver-operating characteristic (ROC) curves were generated for selected variables. The statistical software SPSS Statistics 21.0 (IBM, Madrid, Spain) package and the R software (http://cran.r-project.org) were used for analysis. For generation of chord diagrams, the Circos Table Viewer was employed (http://mkweb.bcgsc.ca).

## Results

### Study population and informative rate

Table 1 summarizes the patients’ pathological and clinical characteristics. The study included 98 consecutive patients with PCa who had undergone radical prostatectomy by open surgery. Urological history and complementary examinations were as follows: prostate volume measured by transrectal ultrasound grade and stage of the biopsy taken from the tumors determined in accordance with the ISUP GG criteria and TNM classification, respectively [22]. With respect to BMI, 51.6% of the patients were overweight, 26.7% were obese and 21.7% were classified as lean. PSA was measured as per standard clinical practice and results showed that 61.8% of patients had levels between 4 and 10 ng/mL, whereas 30.9% had levels above 10 ng/mL. Regarding ISUP GG criteria, 63.3% of the patients were classified as low-risk (group I and II) and 36.7% were high-risk (group III, IV and V). From the 93 patients with clinical-tumoral stage T2, 25.5% were upgraded after surgery; then, 70.4% were classified as ≤ T2 and the remaining 29.6% were classified as T3 or T4.

**Table 1:** Clinical and pathological characteristics of the cohort

	Mean ± SD	N
Age (years)	63.5 ± 6.5	98
Prostatic volume (c.c)	44.4 ± 22.4	98
Testosterone (nmol/L)	13.9 ± 4.9	98
Total PSA (ng/mL)	9.7 ± 8.20	98

		N (%)
BMI (kg/m^2^)	< 25	21 (21.7)
	25–29.99	50 (51.6)
	≥ 30	26 (26.7)
Total PSA (µg/L)	< 4	7 (7.2)
	4–10	60 (61.9)
	> 10	30 (30,9)
ISUP GG		
Low risk	Group I	38 (38,8)
	Group II	24 (24,5)
High risk	Group III	19 (19.3)
	Group IV	10 (10.2)
	Group V	7 (7.2)
T pathological stage	≤ T2a	69 (70.4)
	T3, T4	29 (29,6)
No pathological stage	NX	53 (55.8)
	N0	36 (37.9)
	N1	6 (6.3)

*N* number of patients, *SD* standard deviation, *BMI* body mass index, *ISUP GG* ISUP Grade Groups based on the Gleason score as follows: (Gleason score ≤ 6 group I; 3 + 4=7 group II; 4 + 3=7 group III; 4 + 4=8 group IV; and 9–10 group V); T-stage tumor category; N lymph node category

### Correlation analysis of biofluid biomarkers with clinical and metabolic parameters of the study population

**Fig. 1:**
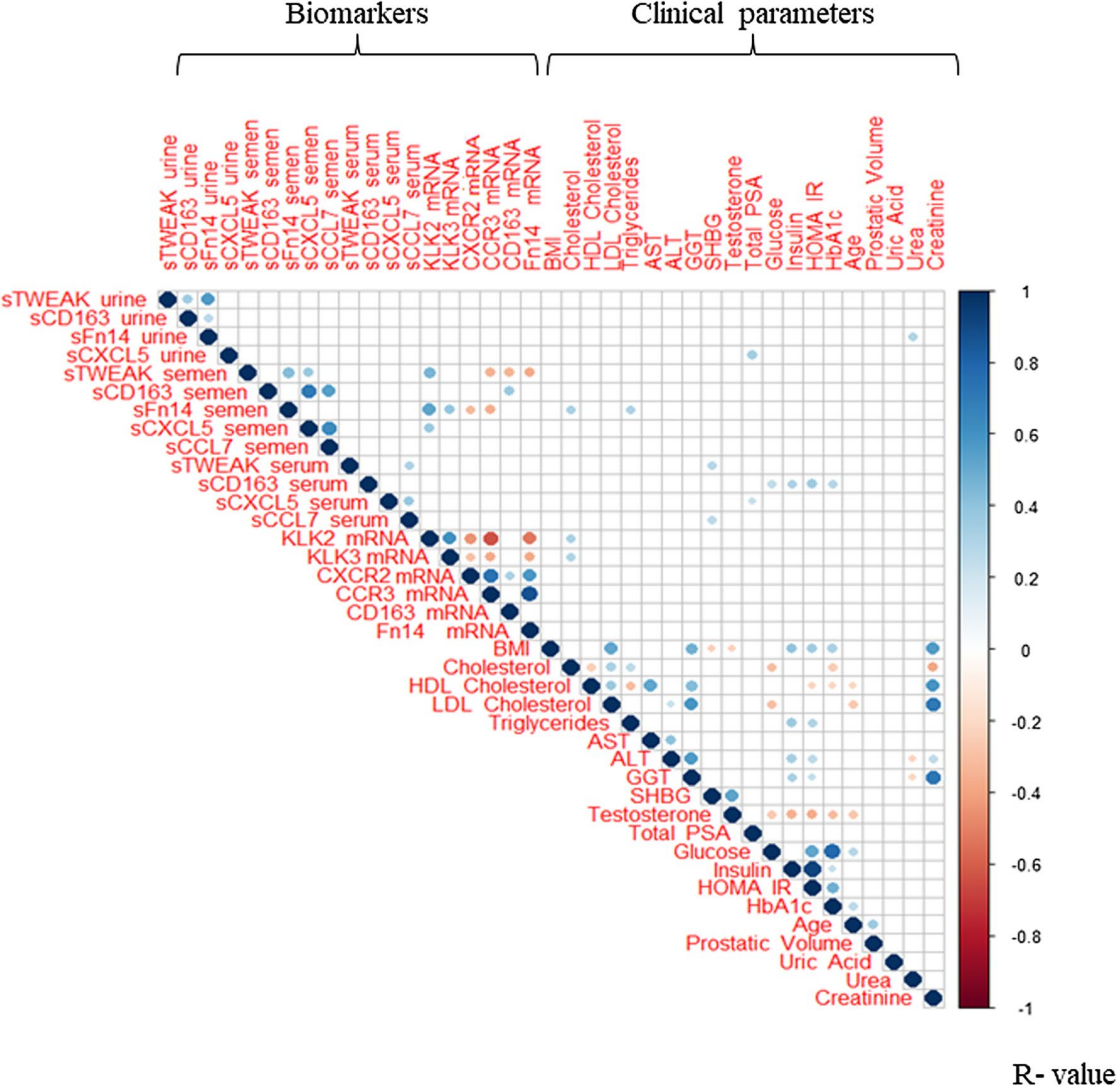
Spearman correlation matrix. Correlation map plotted using significance levels for Spearman test performed with relevant clinical and biomarker data from all studied patients. Positive correlations are displayed in graded blue colors and negative correlations in graded red colors. Correlations with *p*-value ≥ 0.05 are considered as insignificant and are left blank. Color intensity and the size of the circle are proportional to the correlation coefficients. In the right side of the correlogram, the color legend shows the correlation coefficients and the corresponding colors. *BMI* body mass index, *HOMA-IR* homeostatic model assessment for insulin resistance, *HbA1c* hemoglobin A1c, *HDL* high-density lipoprotein, *LDL* low-density lipoprotein, *AST* aspartate aminotransferase, *ALT* alanine aminotransferase, *GGT* gamma glutamyltransferase, *SHBG* sex hormone-binding globulin, *PSA* prostate-specific antigen, *TWEAK* tumor necrosis factor-like weak inducer of apoptosis, *CD163* cluster of differentiation 163, *Fn14* fibroblast growth factor-inducible 14, *CXCL5* C-X-C motif chemokine 5, *CCL7* chemokine (C-C motif) ligand 7, *CXCR2* C-X-C motif chemokine receptor 2, *CCR3* C-C chemokine receptor type 3, *KLK2* kallikrenin-2 gene, *KLK3* Kallikrenin-3 gene

Analysis of correlation by Spearman’s bivariate correlation test, considering only the significant associations, is shown in Fig. 1. We found a positive association between urine sTWEAK levels and urine sFn14 (r = 0.721, p < 0.001) and sCD163 (r = 0.417, p ˂ 0.001) levels, whereas sCXCL5 urine levels correlated with serum total PSA levels (r = 0.250, p = 0.018). Similarly, sFn14 in urine was positively related to urea levels (r = 0.299, p = 0.007). No other associations were observed between biomarkers in urine and those in serum or semen.

Regarding the associations of biomarkers in semen, we found that semen plasma sTWEAK levels were positively associated with sFn14 (r = 0.405, p = 0.003), sCXCL5 (r = 0.362, p = 0.009) and *KLK2* mRNA levels in semen cell sediment (r = 0.552, p < 0.001), and negatively associated with *CCR3*, *CD163* and *Fn14* mRNA levels in semen cell sediment (r = -0.404, p = 0.003; r = − 0.333, p = 0.041; r = − 0.413, p < 0.001, respectively). Also, sCD163 levels were positively associated with sCXCL5 (r = 0.502, p < 0.001) and sCCL7 levels (r = 0.601, p < 0.001), and a weak positive association was found with its mRNA level in semen cell sediment (r = 0.328, p = 0.034). The levels of sFn14 were associated positively with *KLK2* and *KLK3* mRNA levels in semen cell sediment (r = 0.556, p < 0.001; r = 0.494, p < 0.001, respectively) and with serum lipid markers including cholesterol and triglycerides (r = 0.346 p = 0.012; r = 0.348 p = 0.016, respectively), and negatively associated with *CXCR2* and *CCR3* mRNA levels in semen cell sediment (r = − 0.378, p = 0.012; r = − 0.473, p = 0.001). Finally, sCXCL5 levels in semen were positively associated with sCCL7 levels (r = 0.563 p < 0.001) and with *KLK2* mRNA levels in semen cell sediment (r = 0.447, p = 0.003).

Analysis of biomarkers in serum showed that sTWEAK and sCXCL5 were positively associated with sCCL7 levels (r = 0.309, p = 0.006; r = 0.371, p = 0.001, respectively). Also, sCD163 was associated with glycemic parameters such as fasting glycemia (r = 0.243, p = 0.031), insulin (r = 0.365, p = 0.001), HOMA-IR index (r = 0.387, p < 0.001) and HbA1_c_ (r = 0.271, p = 0.016). No associations between biomarkers in serum and semen were observed.

Regarding the mRNA levels of the selected biomarkers in semen cell sediment, we observed a positive association between *KLK2* and *KLK3* (r = 0.603, p < 0.001), whereas *KLK2* was negatively associated with *CXCR2, CCR3* and *Fn14* (r = − 0.442, p = 0.003; r = − 0.635, p < 0.001; r = − 0.520, p < 0.001, respectively). Additionally, *CXCR2* was strongly associated with *CCR3* and *Fn14* (r = 0.918, p < 0.001; r = 0.725, p < 0.001). Of note, cholesterol serum levels had a weak positive association with mRNA levels of *KLK2* and *KLK3* (Fig. 1).

### Univariate analysis of biofluid biomarkers and clinical and metabolic parameters

We next compared these variables in patients classified according to ISUP group: low-risk (group I and II) and high-risk (group III, IV and V). Differences in clinical and metabolic characteristics and biomarker profiles in semen serum, urine and semen cell sediment (gene expression) were recorded in both groups (Table 2).

**Table 2:** Anthropometric, clinical and biochemical variables classified according to ISUP GG patient classification

	ISUP GG classification		
	Low risk (group I and II)	High risk (group III, IV and V)	*p*-value
	Mean ± SD	Mean ± SD	
Anthropometric parameters			
Age (years)	62.82 ± 6.8	64.55 ± 5.82	0.320
BMI (kg/m^2^)	28.23 ± 4.05	27.94 ± 3.54	0.665
Prostatic volume (cc)	45.71 ± 25.96	43.5 ± 19.52	0.716
Glycemic profile			
Glucose (mmol/L)	5.90 ± 1.23	6.65 ± 2.35	0.050
Insulin (pmol/L)	95.42 ± 60.84	89.73 ± 48.41	0.847
HOMA-IR	3.75 ± 2.92	3.92 ± 2.76	0.795
HbA1c (%)	5.77 ± 0.67	6.01 ± 0.86	0.152
Lipid profile			
Cholesterol (mmol/L)	5.02 ± 1.06	4.93 ± 1.01	0.609
HDL cholesterol (mmol/L)	1.49 ± 0.71	1.35 ± 0.39	0.484
LDL cholesterol (mmol/L)	3.23 ± 1.29	2.84 ± 0.88	0.148
Triglycerides (mmol/L)	1.40 ± 0.74	1.63 ± 0.99	0.526
Hepatic profile			
AST (µkat/L)	0.38 ± 0.19	0.32 ± 0.07	0.104
ALT (µkat/L)	0.40 ± 0.21	0.36 ± 0.11	0.816
GGT (µkat/L)	0.72 ± 0.88	0.68 ± 0.50	0.783
Renal profile			
Uric acid (µmol/L)	373.57 ± 88.63	463.39 ± 559.75	0.595
Urea (mmol/L)	13.99 ± 3.34	14.44 ± 5.40	0.922
Creatinine (μmol/L)	83.98 ± 1.77	77.79 ± 2.19	0.187
Hormonal profile			
SHBG (nmol/L)	46.64 ± 50.65	38.93 ± 16.71	0.756
Testosterone (nmol/L)	14.22 ± 4.62	12.88 ± 5.80	0.158
Tumoral markers			
Total PSA (μg/L)	7.64 ± 4.42	13.14 ± 11.47	0.003
Biofluid biomarker profile			
Semen cytokines (pg/mg of total protein)			
sTWEAK	989.07 ± 624.08	550.54 ± 380.25	0.003
sCD163	1449.03 ± 2060.7	1392.09 ± 1265.89	0.391
sFn14	645.31 ± 434.45	609.99 ± 413.28	0.781
sCXCL5	360.23 ± 376.07	212.72 ± 240.72	0.179
sCCL7	3.09 ± 3.34	3 ± 2.69	0.767
Serum cytokines (pg/mL)			
sTWEAK	1118.74 ± 344.32	1331.08 ± 830.51	0.225
sCD163	346.68 ± 286.15	349.46 ± 276.29	0.976
sCXCL5	1234.89 ± 630.15	1346.42 ± 703.23	0.439
sCCL7	2.6 ± 17.62	23.49 ± 107.42	0.508
Urine cytokines (pg/mg of creatinine)			
sTWEAK	199.62 ± 257.43	128.24 ± 151.58	0.723
sCD163	166.07 ± 804.1	1173.09 ± 3368.52	0.401
sFn14	182.5 ± 287.53	159.69 ± 236.24	0.479
sCXCL5	132.97 ± 142.43	161.98 ± 165.27	0.423
Semen cell sediment gene expression (arbitrary units, 2^−∆∆Ct^)			
*KLK2*	1.12 ± 0.41	0.82 ± 0.45	0.050
*KLK3*	1.16 ± 0.75	1.02 ± 0.74	0.529
*CXCR2*	2.68 ± 3.6	4.03 ± 3.7	0.089
*CCR3*	2.53 ± 3.23	6.31 ± 8.1	0.069
*CD163*	6.37 ± 6.57	5.68 ± 5.54	0.796
*Fn14*	4.15 ± 2.82	8.52 ± 7.69	0.077

BMI, Body mass index; cc, centiliters; HOMA-IR, homeostatic model assessment for insulin resistance; HbA1c, hemoglobin A1c; HDL, high-density lipoprotein; LDL, low-density lipoprotein; AST, aspartate aminotransferase; ALT, alanine aminotransferase; GGT, gamma glutamyltransferase; SHBG, sex hormone-binding globulin; PSA, prostate specific antigen; TWEAK, tumor necrosis factor-like weak inducer of apoptosis; CD163, cluster of differentiation 163; Fn14, fibroblast growth factor-inducible 14; CXCL5, C-X-C motif chemokine 5; CCL7, chemokine (C-C motif) ligand 7; *CXCR2*, C-X-C motif chemokine receptor 2; *CCR3*, C-C chemokine receptor type 3; *KLK2*, Kallikrenin-2 gene; *KLK3*, Kallikrenin-3 gene

**Fig. 2:**
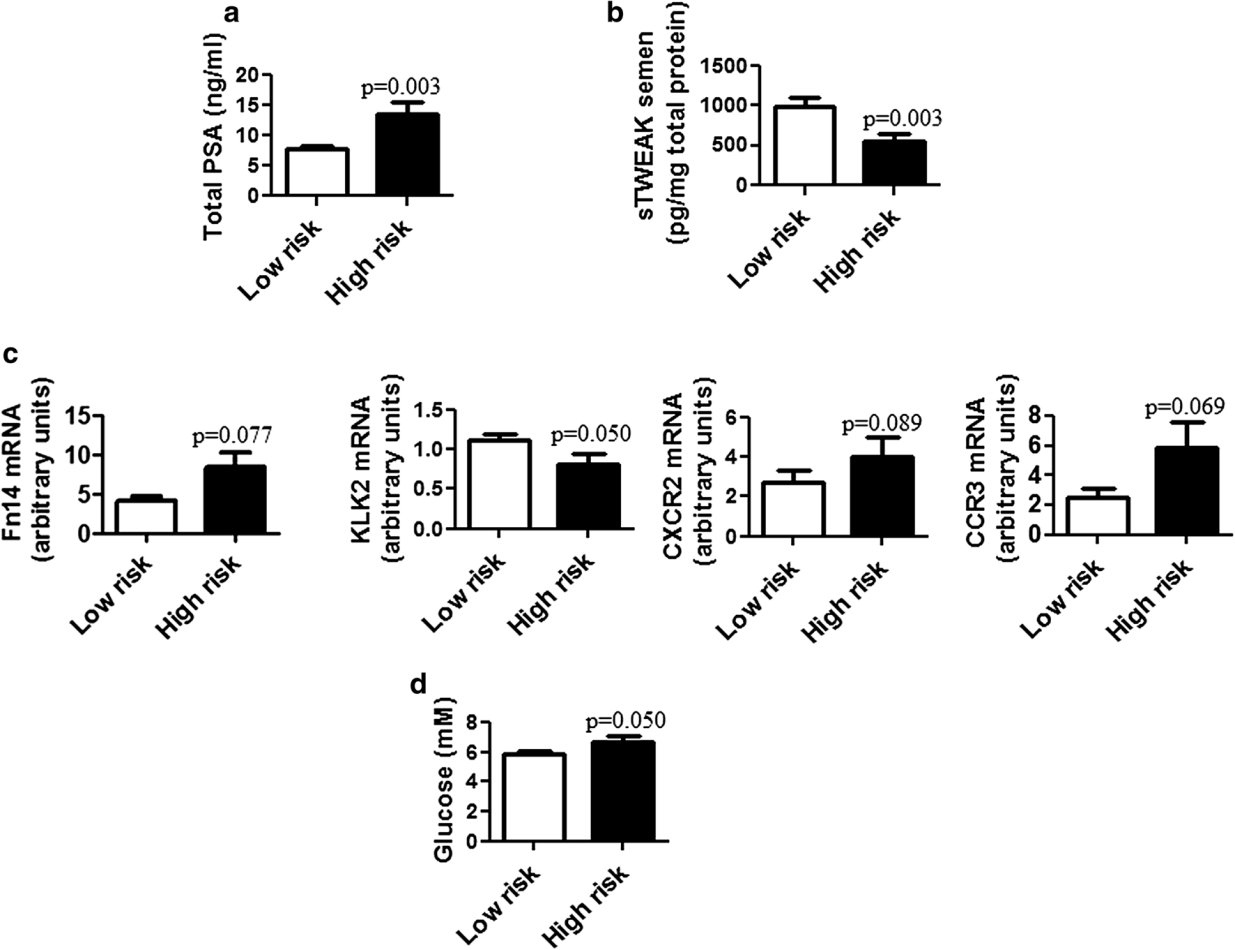
Biomarker panel for prediction of prostate cancer aggressiveness. Box plot representing significant changes of biomarker levels in different biofluids: **a** Serum total PSA, **b** sTWEAK semen levels, **c** mRNA levels in semen cell sediment of: *Fn14*, *KLK2*, *CXCR2* and *CCR3*, and **d** fasting glucose levels. Values of each biomarker are expressed as mean ± SD. Differences between patients according to Gleason stage coded into two categories: low-risk ISUP GG (group I and II) or high-risk ISUP GG (group III, IV and V) were tested with the Mann–Whitney U test for non-normally distributed data. *p*-values less than 0.05 were considered statistically significant

As expected, univariate analysis showed that total serum PSA was significantly higher in the high-risk group than in the low-risk group (p = 0.003) (Fig. 2a). sTWEAK levels in semen were significantly lower in the high-risk group than in the low-risk group (p = 0.003) (Fig. 2b). Regarding gene expression levels, we found higher mRNA expression of *KLK2* in the low-risk group than in the high-risk group (p = 0.05). Of note, there was a trend for higher *Fn14*, *CXCR2* and *CCR3* gene expression levels in semen cell sediment in the high-risk group relative to the low-risk group, but this did not reach statistical significance (p = 0.077, p = 0.089, p = 0.069, respectively) (Fig. 2c). No significant differences were detected between compared groups regarding anthropometric parameters, lipid, hepatic, renal and hormonal profiles. By contrast, serum glucose levels were significantly higher in the high-risk group than in the low-risk group (p = 0.05) (Fig. 2d).

**Fig. 3:**
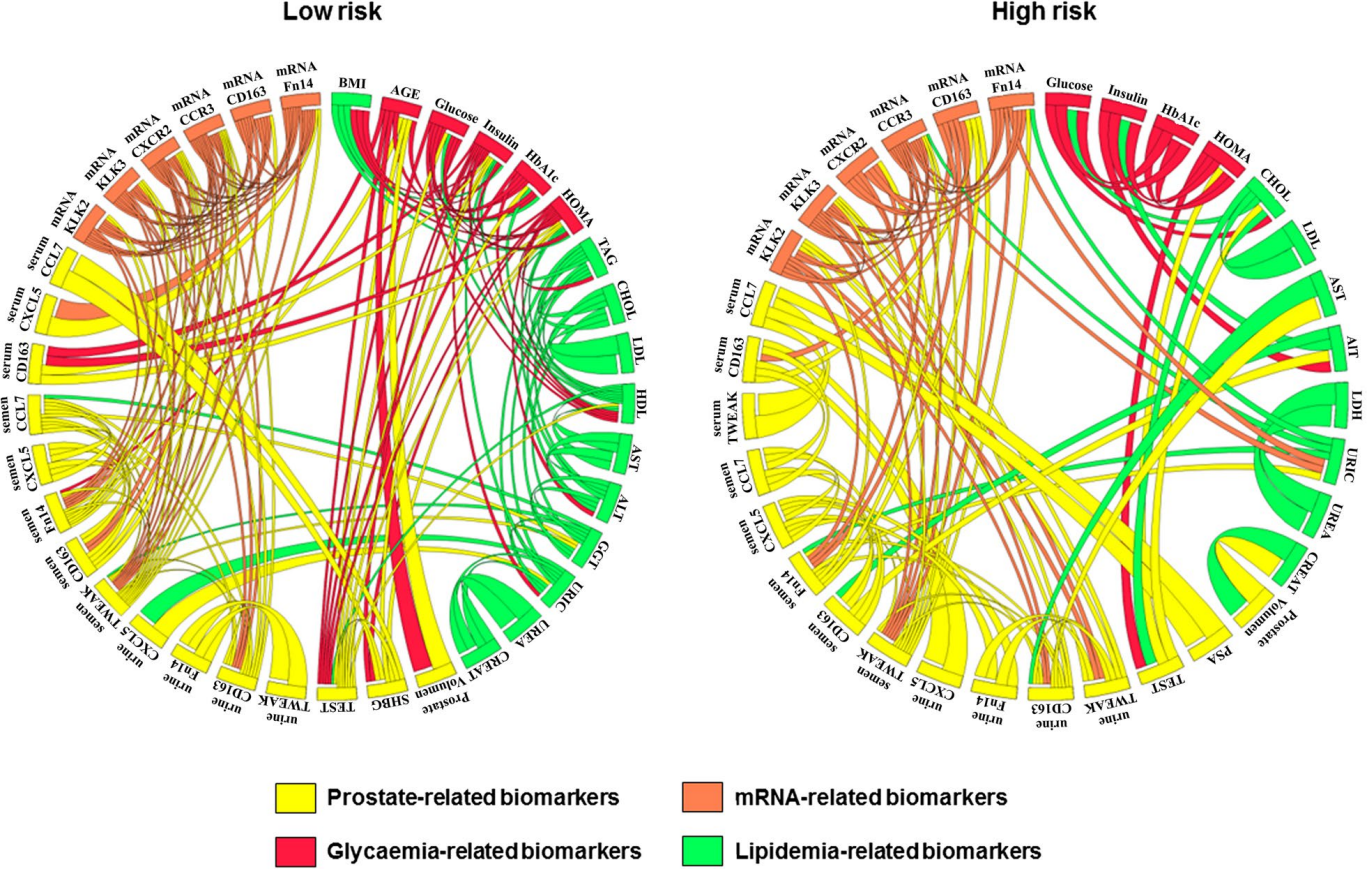
Chord diagram showing differences in correlations between variables across individuals showing differential ISUP GG risk: low-risk ISUP GG (group I and II) and high-risk ISUP GG (group III, IV and V). Segments in circles indicate variables analyzed (see below for abbreviations employed), chords linking variables indicate significant correlations (at least *p *< 0.05) according to Spearman rank correlations, while chord thickness is directly proportional to correlation coefficient. For clarity, only positive correlations are shown. *TEST* testosterone, *CREAT* creatinine, *HOMA-IR* homeostatic model assessment for insulin resistance, *HbA1c* hemoglobin A1c, *HDL* high-density lipoprotein, *LDL* low-density lipoprotein, *CHOL* cholesterol, *AST* aspartate aminotransferase, *ALT* alanine aminotransferase, *GGT* gamma glutamyltransferase, *PSA* prostate-specific antigen, *TWEAK* tumor necrosis factor-like weak inducer of apoptosis, *CD163* cluster of differentiation 163, *Fn14* fibroblast growth factor-inducible 14, *CXCL5* C-X-C motif chemokine 5, *CCL7* chemokine (C-C motif) ligand 7, *CXCR2* C-X-C motif chemokine receptor 2, *CCR3* C-C chemokine receptor type 3, *KLK2* kallikrenin-2 gene, *KLK3* kallikrenin-3 gene

Notably, the evaluation of correlations among anthropometric and metabolic parameters in individuals with less aggressive tumors (group I and II) compared with those with more aggressive tumours (groups III, IV and V) showed clear differences between both groups of subjects (Fig. 3).

### Potential relevance of biofluid biomarkers in the stratification of PCa aggressiveness

**Fig. 4:**
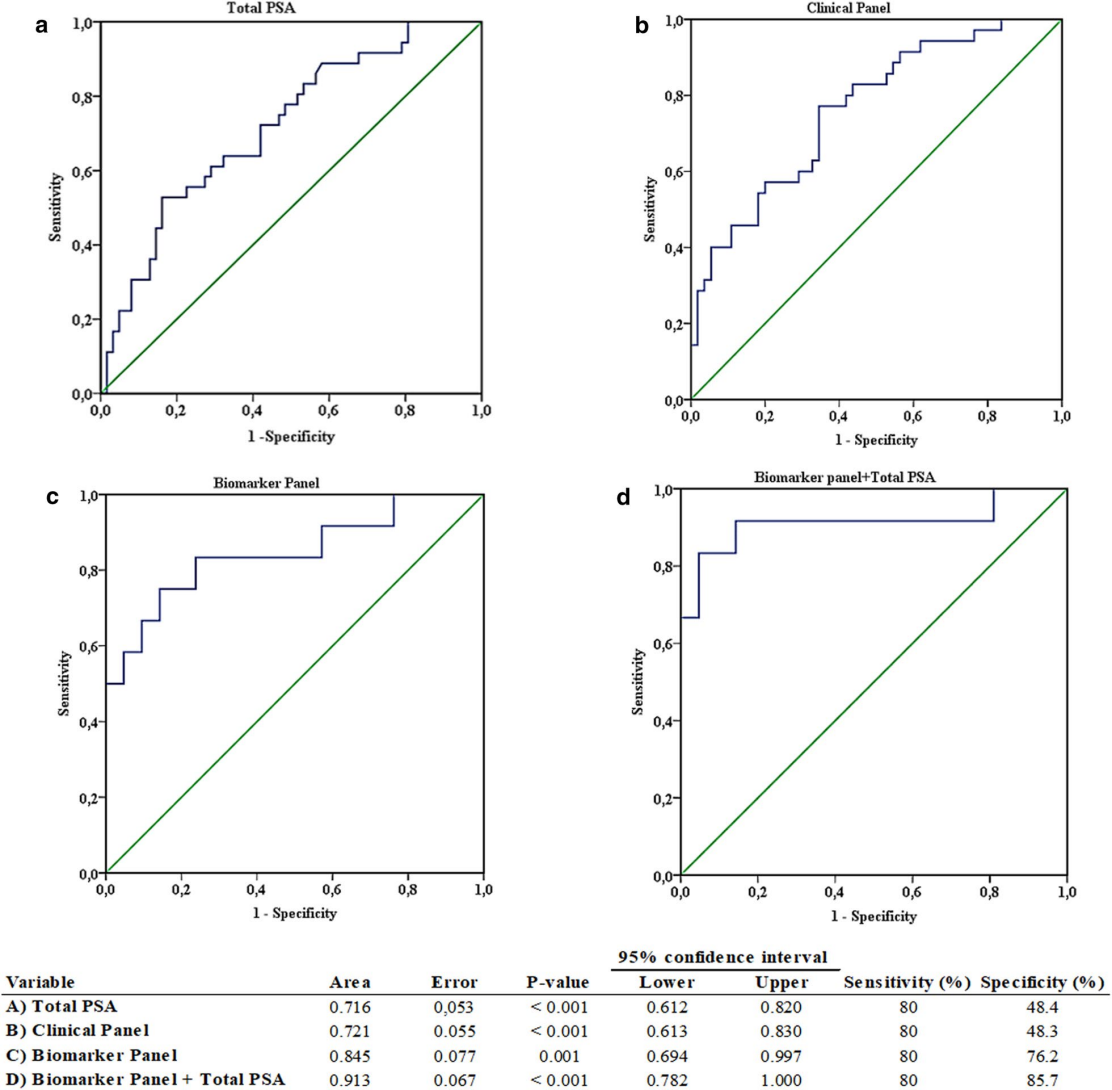
Biomarker models for prediction of prostate cancer aggressiveness. Receiver-operating characteristic curve (ROC) analysis as assessed according to low-risk ISUP GG (group I and II) and high-risk ISUP GG (group III, IV and V). **a** Total PSA, **b** Clinical panel (Age, T-classification, total PSA), **c** Biomarker panel (sTWEAK in semen, glucose levels in serum and mRNA levels of *Fn14*, *KLK2*, *CXCR2* and *CCR3* in semen cell sediment), **d** Biomarker panel in combination with total PSA

To evaluate the performance of a selected biomarker panel (Fig. 2) as a predictor of PCa aggressiveness, we performed ROC analysis for total PSA in serum, one of the primary differentiator variables of patients stratified according to ISUP GG risk categories. The diagnostic accuracy of this signature had an area under the curve (AUC) of 0.716 (95% confidence interval [CI] 0.612–0.820), with 80% sensitivity and 48.4% specificity, and could correctly classify 68.4% of patients (Fig. 4a).

When we used a panel of routinely used non-invasive clinical variables for patient classification in a logistic regression model, by combining age, clinical stage (T-classification) and total PSA levels in serum, we obtained a model that allowed the correct identification of 72.9% of patients. The ROC curve obtained using the non-invasive clinical panel had an AUC of 0.721 (95% CI 0.613–0.830), with 80% sensitivity and 48.3% specificity (Fig. 4b).

As we observed differences in some of the abovementioned biomarkers between ISUP GG risk categories, we tested the ability of a selected biomarker panel to predict PCa aggressiveness. A logistic regression model helped to identify the following determinants: sTWEAK in semen, fasting glycemia and the expression levels of *Fn14*, *KLK2*, *CXCR2* and *CCR3* in semen cell sediment. This analysis could correctly identify 81.1% of patients according to histologic Gleason aggressiveness. The ROC curve obtained with this non-invasive biomarker panel had an AUC of 0.845 (95% CI 0.694–0.997), with 80% sensitivity and 76.2% specificity, enhancing the specificity of these parameters as compared with the aforementioned non-invasive classical clinical panel (Fig. 4c).

We also assessed the power of classification by combining the selected non-invasive biomarker panel plus total PSA serum levels, and the resulting logistic regression model outperformed the other models, with patient identification of 90.9%, an AUC of 0.913 (95% CI 0.782–1), and with 80% sensitivity and 85.7% specificity (Fig. 4d).

## Discussion

There is a desperate need to correctly identify patients with PCa and aggressive disease to guide treatment decisions and to avoid unnecessary prostate procedures. Biomarkers are used extensively in clinical practice to predict the presence of PCa and to distinguish aggressive from indolent forms [24]. While circulating serum total PSA is used extensively for general screening to diagnose the presence of PCa, new biomarkers are needed for the implementation of more personalized diagnosis and management of PCa.

To identify new biomarkers for PCa in liquid biopsies, in the present work we measured the circulating levels of sTWEAK and its receptors sFn14 and sCD163, in addition to two chemokines, CXCL5 and CCL7, all of which are closely related to prostate tumorigenesis [17, 19] but never analyzed in human PCa liquid biopsies. Additionally, we evaluated the expression of their related receptors (*Fn14*, *CD163*, *CXCR2*, and *CCR3*) in semen cell sediment as well as gene expression of the PSA-related genes *KLK2* and *KLK3,* as possible biomarkers for PCa aggressiveness.

In the context of cancer, circulating levels of sTWEAK have previously been described as a biomarker in head and neck cancer, where low serum levels of sTWEAK were found to be related to reduced survival rate [25, 26], pointing to sTWEAK levels as a possible non-invasive biomarker in this patient group. To the best of our knowledge, no study has reported on the potential of sTWEAK as a PCa biomarker; however, it is important to consider that a pro-tumorigenic role of the TWEAK/Fn14 axis has previously been evaluated in vitro in several PCa cell types [8, 10, 27, 28].

Bivariate correlation analysis showed that semen levels of sTWEAK and sFn14 were positively related and also correlated with *KLK2* mRNA expression in semen cell sediment. *KLK2* is a member of the Kallikrein family of secreted serine proteases, which are associated with prostatic tissue [29]. *KLK2* is highly expressed in prostate tumor cells and might be a prognostic marker for PCa risk [30]. Also, the expression of *KLK2* is highly correlated with that of *KLK3* [29], which allowed us to determine the prostate epithelial cell content in semen [3], suggesting that the positive correlation of sTWEAK and sFn14 in semen could be directly related to prostate cell epithelium.

Interestingly, a strong positive association was observed between sCD163 and sCXCL5 and sCCL7 in semen. All of these molecules are known to be expressed mainly in macrophages [31]. Additionally, sCD163 is specifically related to alternative “M2-type” macrophages, which are abundantly found in cancer [32], whereas CXCL5 protein expression levels are concordant with prostate tumor progression. Furthermore, both CD163 and CXCL5 are highly associated with inflammatory infiltrates, which are frequently detected in the lumen of malignant prostate glands [33].A relationship between the TWEAK/Fn14 axis and CD163 was also found in urine.

In serum, sTWEAK levels positively correlated with sCCL7 and sCXCL5, and it is known that high expression of *CXCL5* is closely related to cancer progression [34–36], which is in line with the association we observed in our cohort between PSA levels and sCXCL5 in urine. Of particular note is the association found between serum levels of sCD163 and glycemia-related variables (insulin, HOMA-IR index and fasting glycemia), corroborating previous findings for this biomarker in the context of diabetes and altered glucose metabolism [37, 38].

In serum, sTWEAK levels were unchanged between the high- and low-risk patient groups. Serum sTWEAK levels are reduced in obesity [39]. In our cohort, 51.6% of the patients were overweight and 26.7% were obese, but no association was observed between sTWEAK serum levels and BMI. However, we found that semen levels of sTWEAK were significantly altered according to Gleason histologic aggressiveness. The reduction in sTWEAK levels in semen in high-risk patients was accompanied by a tendency for an increase in *Fn14* mRNA levels in semen cell sediment. This finding might indicate an active process of ligand–receptor interaction in this microenvironment, in which the ligand–receptor complex must be internalized, thereby reducing ligand availability and favoring the process of proliferation and migration, as described in PCa cells in vitro mediated by the TWEAK/Fn14 axis [10, 28].

Interestingly, the mRNA expression levels of *CCR3* and *CXCR2* in semen cell sediment showed a similar behavior to *Fn14* mRNA in the ISUP high-risk patients, which might indicate a higher activity of the respective ligands CCL7 and CXCL5 at this site, although no significant changes were found for these soluble chemokines in our patients between high- and low-risk groups. We observed a decreased expression of *KLK2* in semen cell sediment in high-risk patients, whereas PSA levels were higher. Although a positive relationship was expected, we do not have a clear explanation for this finding. Of note, it has been reported that *KLK2* expression represents only 50% of total PSA [40].

Concerning the profiles of metabolic and biochemical parameters (lipidic, hepatic, renal and hormonal), we found differences only in the levels of serum glucose between low- and high-risk PCa patients in our cohort, with elevated levels of glucose in the latter group. This is consistent with a previous finding of an association between serum glucose levels and disease outcome in colorectal, breast, bladder, pancreatic and PCa patients [41]. By contrast, correlation patterns between anthropometric and metabolic variables across individuals with high-risk and low-risk were markedly different, as demonstrated by chord diagram analysis, pointing to an intrinsic metabolic behaviour in these groups. Serum cholesterol may be related to PCa biology [42, 43], in this sense, we observed an association between cholesterol levels and *KLK3* and *KLK2* gene expression, both related to PSA protein levels.

While PSA provides a sensitive marker for PCa diagnosis, it is not only confined to PCa, hence it cannot be used alone as a diagnostic PCa biomarker [44]. In our study, ROC curve analysis using PSA alone had no advantage in its predictive ability, either alone or combined with a classical clinical panel (age, T-classification, total PSA). These results prompted us to question whether we could establish another suitable non-invasive biomarker panel for PCa aggressiveness by inputting a combination of the new biomarkers studied. Indeed, ROC curve analysis demonstrated that this new non-invasive biomarker panel comprising sTWEAK in semen, glucose levels in serum and mRNA levels of *Fn14*, *KLK2*, *CXCR2* and *CCR3* in semen cell sediment had a better AUC than PSA and even the classical non-invasive clinical panel (age, T-classification, total PSA) (AUCs of 0.845, 0.716 and 0.721, respectively), and could classify patients according to PCa aggressiveness with higher specificity when compared with either total PSA classification or when using the non-invasive classical clinical panel (specificity of 76.2%, 48.4% and 48.3%, respectively). Moreover, the combination of the above biomarker panel to supplement total serum PSA improves the AUC stratification (91.3%) of tumor aggressiveness, as classified by ISUP high-risk or low-risk. This new panel (biomarker panel plus total serum PSA) could be used to correctly classify tumor aggressiveness in 90.9% patients with higher specificity (85.7%). Although our findings are encouraging, a larger cohort will be needed to support the biomarker panel for positive diagnosis.

## Conclusions

A biofluid signature comprising sTWEAK in semen, serum fasting glycemia and the mRNA levels of *Fn14*, *KLK2*, *CXCR2* and *CCR3* in semen cell sediment, combined with total serum PSA levels, represents a non-invasive biomarker panel with high negative predictive value that can be used to classify PCa aggressiveness.

## Data Availability

All data generated or analyzed during this study are included in this published article.

## Abbreviations

BMI: body mass index
c.c: centiliters
HOMA-IR: Homeostatic Model Assessment for Insulin Resistance
HbA1c: hemoglobin A1c
HDL: high-density lipoprotein
LDL: low-density lipoprotein
AST: aspartate aminotransferase
ALT: alanine aminotransferase
GGT: gamma glutamyltransferase
SHBG: sex hormone binding globulin
PSA: prostate-specific antigen
TWEAK: tumor necrosis factor-like weak inducer of apoptosis
CD163: cluster of differentiation 163
Fn14: fibroblast growth factor-inducible 14
CXCL5: C-X-C motif chemokine 5
CCL7: chemokine (C-C motif) ligand 7
*CXCR2*: C-X-C Motif Chemokine Receptor 2
*CCR3*: C-C chemokine receptor type 3
*KLK2*: kallikrein-2
*KLK3*: kallikrein-3
